# Cardioprotective Activity of Selected Polyphenols Based on Epithelial and Aortic Cell Lines. A Review

**DOI:** 10.3390/molecules25225343

**Published:** 2020-11-16

**Authors:** Michał Otręba, Leon Kośmider, Jerzy Stojko, Anna Rzepecka-Stojko

**Affiliations:** 1 Department of Drug Technology, Faculty of Pharmaceutical Sciences in Sosnowiec, Medical University of Silesia in Katowice, Jednosci 8, 41-200 Sosnowiec, Poland; annastojko@sum.edu.pl; 2 Department of General and Inorganic Chemistry, Faculty of Pharmaceutical Sciences in Sosnowiec, Medical University of Silesia in Katowice, Jagiellonska 4, 41-200 Sosnowiec, Poland; leon.kosmider@gmail.com; 3 Department of Toxicology and Bioanalysis, Faculty of Pharmaceutical Sciences in Sosnowiec, Medical University of Silesia in Katowice, Ostrogorska 30, 41-200 Sosnowiec, Poland; jstojko@sum.edu.pl

**Keywords:** polyphenols, endothelial cells, aortic cells, cardioprotective activity

## Abstract

Polyphenols have recently gained popularity among the general public as products and diets classified as healthy and containing naturally occurring phenols. Many polyphenolic extracts are available on the market as dietary supplements, functional foods, or cosmetics, taking advantage of clients’ desire to live a healthier and longer life. However, due to the difficulty of discovering the in vivo functions of polyphenols, most of the research focuses on in vitro studies. In this review, we focused on the cardioprotective activity of different polyphenols as possible candidates for use in cardiovascular disease therapy and for improving the quality of life of patients. Thus, the studies, which were mainly based on endothelial cells, aortic cells, and some in vivo studies, were analyzed. Based on the reviewed articles, polyphenols have a few points of action, including inhibition of acetylcholinesterase, decrease in reactive oxygen species production and endothelial tube formation, stimulation of acetylcholine-induced endothelium-derived mediator release, and others, which lead to their cardio- and/or vasoprotective effects on endothelial cells. The obtained results suggest positive effects of polyphenols, but more long-term in vivo studies demonstrating effects on mechanism of action, sensitivity, and specificity or efficacy are needed before legal health claims can be made.

## 1. Introduction

The three main groups of compounds containing one or more polyphenol groups [1] are known: flavonoids (flavonols [2,3], anthocyanins [2,3], flavan-3-ols (catechins) [2,3], flavones [2,3], and chalcones [2]), nonflavonoids (stilbenoids [2,3], curcumin [3], coumarins [2,3], and neolignans [2]), and phenolic acids (ellagic acid [3], tannic acid [2,3], gallic acid [3], and caffeic acid [3]). Resveratrol, pterostilbene, piceatannol, and gnetol belong to the stilbenoids group [4]. Polyphenolic compounds are natural plant products that are responsible for fruit color and protection against pathogens [3]. Polyphenols possess antioxidant activity, but they also have the potential to stimulate oxidative stress by free radical production [2]. The antioxidant action of polyphenols is possible due to radical scavenging (OH^•−^ and NO^•−^), metal chelating (Fe^2+^, Cu^2+^, Co^2+^, Ti^3+^, Cr^5+^, and V^2+^), nitro-oxidase (NOX) inhibition, impact on mitochondria (block of the mitochondrial respiratory chain and inhibition of ATPase at the inner mitochondrial membrane), inhibition of xanthine oxidase, and upregulation of antioxidant enzymes (superoxide dismutase, catalase, glutathione peroxidase, and heme oxygenase-1) [3]. Moreover, a lot of important biological activities of polyphenols, such as antiallergenic, anti-inflammatory, antimicrobial, antioxidant, antithrombotic, cardioprotective, and vasodilatory effects, are known. Stilbenes, such as resveratrol, possess antioxidant and anti-inflammatory effects, as well as cardioprotective and platelet antiaggregant activities [2].

The cardioprotective effect of polyphenols is caused by antioxidative, anticoagulation, antiplatelet aggregation; fibrinolysis activity; activation of AMP-activated protein kinase, nitric oxide synthase, and sirtuin 1; inhibition of angiotensin-converting enzyme and phosphate diesterase; and improvement of endothelial cell function [3]. Moreover, flavonoids improve ventricular health [5], insulin resistance, and plasma lipid markers [6]; possess anti-inflammatory effects [5,6]; and lower blood pressure [5] to increase overall vascular health as well as block cholesterol oxidation to decrease LDL level [5], reduce atherosclerosis [6], and finally reduce the risk of cardiovascular disease. A similar effect is observed after resveratrol treatment [5].

One of the major polyphenols that possess anti-inflammatory, neuroprotective, cardioprotective, and chemopreventive activity is quercetin. The quercetin mechanism of action is related to antioxidant properties (donating electrons or chelating metal ions) and also has interaction with proteins and nucleic acids. This is possible since polyphenols penetrate cellular and nuclear membranes and accumulate in the cell nucleus. Moreover, polyphenols have been shown to interact with different components of protein kinases (regulate multiple cell-signaling pathways by the inhibition of the phosphorylation state), estrogen receptors (prevent breast and ovarian cancer), nuclear receptors, various transcription factors (regulate cell-cycle arrest, apoptosis, and survival), plasma proteins and lipoprotein particles (modify the lipoprotein’s physical and biological structure), and enzymes, such as hydrolases, oxidases, and kinases (alter enzyme structure and activity). Furthermore, polyphenols regulate gene expression and modulate signal transduction pathways. It is noteworthy that quercetin, resveratrol, genistein, and curcumin interact with DNA [7]. The polyphenol mechanism of action is well described in several papers [8,9,10].

According to the World Health Organization (WHO) [11] and Eurostat statistics [12] data for 2016, as well as the European Heart Network data for 2017 [13], increase of deaths caused by cardiovascular diseases (coronary heart disease, cerebrovascular disease, peripheral arterial disease, rheumatic heart disease, congenital heart disease, deep vein thrombosis, and pulmonary embolism) is observed globally. Data showing the number of death cases and percentage of deaths are presented in Table 1. It is noteworthy that in some cases, the global death percentage is higher than the number of deaths caused by malignant neoplasm cancer (26.0%) [12].

Based on the 2019 hearth disease and stroke statistics, the American Heart Association informed that in 2016, cardiovascular diseases caused 840,760 deaths, while in 2019, coronary events were expected to occur in about 1,055,000 individuals. Moreover, approximately every 40 s, an American will have a myocardial infarction, and on average, every 40 s, an American will have a stroke [14]. The stroke mortality age-standardized rates per 100,000 population in 2017 in selected countries [15] are presented in Table 2. 

Polyphenols, which possess anti-inflammatory, antimicrobial, antioxidant, antithrombotic, cardioprotective, and vasodilatory activities, may contribute to acquiring new and effective therapies, leading to treatment and/or preventing cardiovascular diseases.

In this review, we focused on the cardioprotective activity of different polyphenols as possible candidates for use in cardiovascular disease therapy and for improving the quality of life of patients. Thus, the analyzed studies are mainly based on endothelial cells and aortic cells, with some in vivo studies.

## 2. Cardioprotective Activity of Polyphenols

### 2.1. Flavonoids

**Figure 1:**
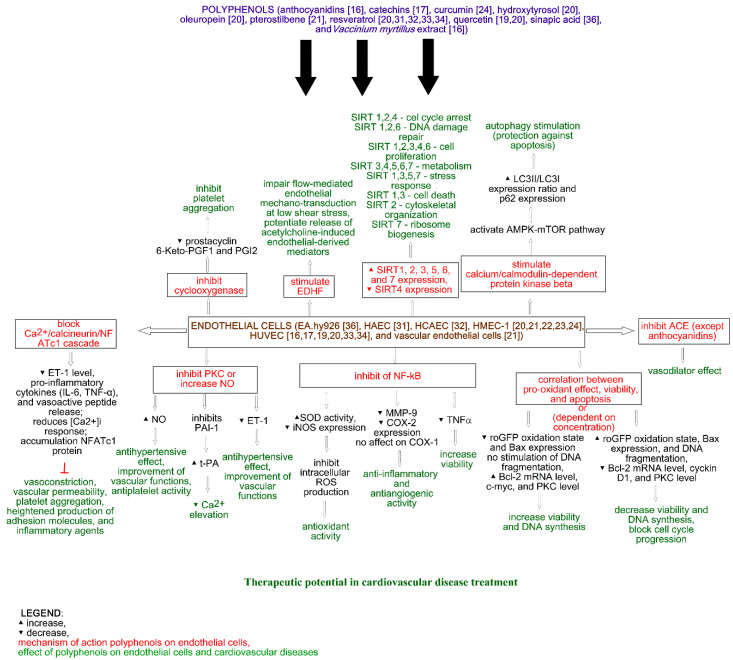
Effect of polyphenols on endothelial cells.

The effect of polyphenols obtained from *Vaccinium myrtillus* on ACE in human umbilical vein endothelial cells (HUVECs) was measured by Persson et al. (2009) [16]. The study showed a decrease in cellular ACE activity after myrtillin chloride treatment. Moreover, the incubation of HUVECs with anthocyanidins (cyanidin, delphinidin, and malvidin) did not affect ACE activity (Figure 1). It is noteworthy that all of the analyzed compounds did not affect cellular viability [16].

**Figure 2:**
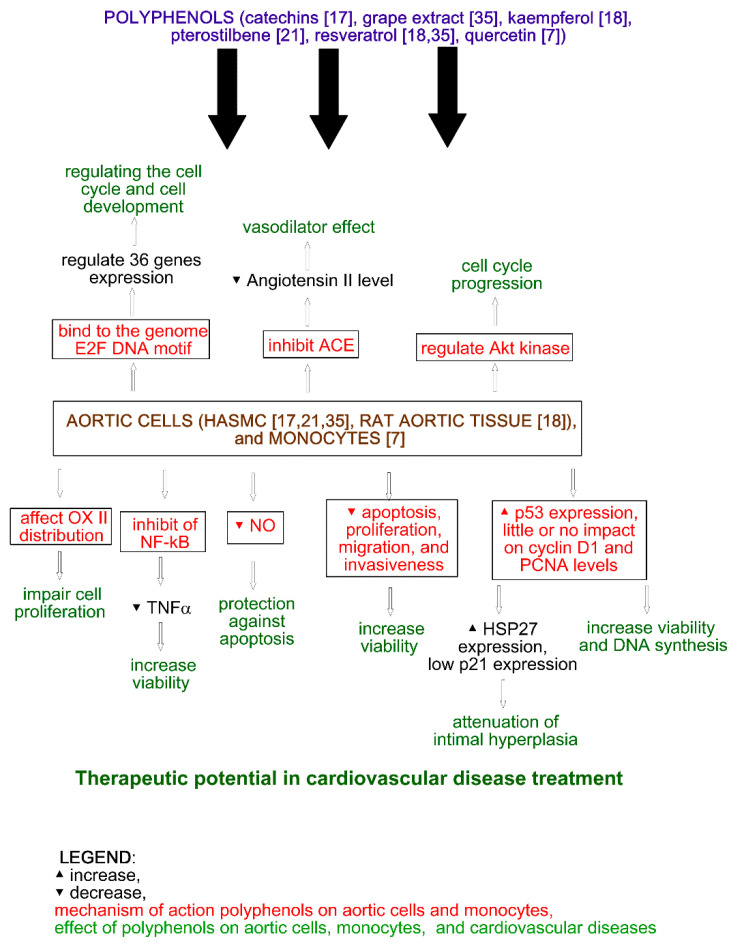
Effect of polyphenols on aortic cells and myocytes.

The effect of catechins on angiogenesis and inflammation was analyzed by Negrão et al. (2013). The analysis of viability showed that catechins at a concentration of 100 µM increase the viability of HUVECs and the human aortic smooth muscle cells (HASMCs) to 165.58 ± 5.04% (*p* ≤ 0.05) and 165.34 ± 31.12% (*p* ≤ 0.05), respectively, and decrease cell proliferation by 33.19 ± 13.56% (*p* ≤ 0.05) in HUVECs and 23.36 ± 8.39% (*p* ≤ 0.05) in HASMCs. Catechin treatment also decreases apoptosis to 53.45 ± 12.88% (*p* ≤ 0.05) and 92.7 ± 4.85% (*p* ≤ 0.05) for HUVECs (1.0 µM) and HASMCs (10 µM), respectively. Moreover, catechins (100 µM) increase the migration of HUVECs and reduce the migration of HASMCs, while at a concentration of 1.0 µM, they decrease the invasiveness of both cell lines. An increase in the number of formations of capillary-like structures was observed after catechin treatment at a concentration of 10 mM. The impact of catechins on HUVECs and HASMCs is shown in Figure 1 and Figure 2, respectively. On the other hand, the incubation of rat aortic rings with catechins (100 µM) did not affect vessel formation. Additionally, catechins did not change microvessel density in the vicinity of the incision area in comparison with the control. The inflammatory modulator assays showed that catechins (100 µM) reduce NFκβ activity, TNFα, and NO levels in HUVECs (by 0%, 58.66 ± 16.20% (*p* ≤ 0.05), and 9.63 ± 0.84% (*p* ≤ 0.05), respectively) and HASMCs (by 38.43 ± 6.01% (*p* ≤ 0.05), 85.46 ± 9.95% (*p* ≤ 0.05), and 5.43 ± 0.81% (*p* ≤ 0.05), respectively). In rat serum, NO level was reduced by 63.47 ± 3.65% (*p* ≤ 0.05). Another in vivo study showed the inhibition of vascular development, and the plugs implanted with catechins possessed angiogenic response [17].

The effect of kaempferol on ACE activity in the aortic tissue of Wistar–Kyoto rats was analyzed by Olszanecki et al. (2009). The authors showed that kaempferol, in contrast to resveratrol, may inhibit angiotensin II formation (Figure 2) by 46% (*p* ≤ 0.01) at a concentration of 100 µmol/L. This may be explained by the presence of the pyran ring with a carbonyl group in the kaempferol structure [18].

The impact of quercetin on endothelin, prostacyclin, and tissue plasminogen activator release from human endothelial cells from human umbilical cord veins (HUVECs) was analyzed by Zhao et al. (1999). The authors showed that the preincubation of the cells with quercetin (5 or 50 mM) for 4 and 24 h decreases endothelin 1 release into the medium. The obtained EC_50_ were 1.54 and 2.78 mM after 4 and 24 h, respectively. It is noteworthy that a 4 h incubation with thrombin increased endothelin 1 level in the medium from 39 ± 8 pg/mL to 136 ± 20 pg/mL. This suggests that the induction of endothelin 1 by thrombin is inhibited by quercetin (Figure 1). Opposite results were noticed for the tissue plasminogen activator—a decrease after 4 h treatment with thrombin (from 4.2 ± 0.4 pg/mL to 3.1 ± 0.3 pg/mL) and an increase after 4 and 24 h treatments with quercetin (EC_50_ = 0.71 and 0.74 mM, respectively, after 4 and 24 h). This suggests that thrombin inhibits the increase of tissue plasminogen release caused by quercetin. The level of prostacyclin 6-Keto-PGF_1β_ increased after thrombin treatment (from 2.3 ± 0.2, 5.8 ± 0.8, 10.5 ± 1.9 pg/mL to 8.09 ± 1.1, 9.1 ± 1.2, 21.2 ± 1.0 pg/mL for 10, 30, and 120 min, respectively) and decreased after quercetin treatment (EC_50_ = 2.1, 2.9, and 0.58 mM, respectively, for 10, 30, and 120 min). This suggests that quercetin inhibits prostacyclin release caused by thrombin treatment [19].

The protective mechanism of red wine (resveratrol and quercetin) and virgin olive oil polyphenols in atherosclerotic vascular disease was analyzed via an in vitro study by Scoditti et al. (2012). Two cell lines were used: HUVEC and human microvascular endothelial cell line (HMEC-1). Analysis of endothelial tube formation in Matrigel showed stimulation of tubelike differentiation of HUVECs and HMECs-1 after 1 h pretreatment with quercetin (1 µmol/L). Quercetin also inhibited HUVEC migration. Moreover, inhibition of MMP-9 specifically expressed in endothelial cells and decrease in COX-2 expression, without affecting the constitutive expression of COX-1 (Figure 1), were observed after quercetin treatment. Finally, the antioxidant capability of the polyphenols was evaluated “as a potential mechanism contributing to the observed MMP-9 and COX-2 inhibition.” The authors observed a decrease of intracellular ROS (Figure 1), which confirmed the antioxidant activity of quercetin [20].

The interaction of quercetin with the genome of the human monocyte THP-1 was analyzed by Atrahimovich et al. 2019. The authors confirmed that quercetin binds to the genome of the cells and has an impact on the expression of 36 genes regulating cell cycle and cell development (Figure 2). The potential DNA motif site binding quercetin is the E2F DNA motif. It is noteworthy that *E2F* encodes transcription factors regulating the cell cycle. Moreover, the authors showed that quercetin treatment changes the expression of cell cycle genes, and quercetin and biotinylated quercetin have a similar effect on cell cycle and development gene expression [7].

### 2.2. Nonflavonoids

The beneficial role of astringin in the setting of ischemia reperfusion of a rat model was confirmed by in vivo studies. The incidence and duration reduction of ventricular tachycardia and fibrillation were observed after astringin treatment. Moreover, a decrease in mortality rate and lactate dehydrogenase levels and an increase of nitric oxide were observed, suggesting the cardioprotective and antiarrhythmic properties of astringin [21].

The hypotensive effect of butein was measured in vivo by Kang et al. (2003). The authors observed in anesthetized normotensive rats that butein decreases arterial blood pressure, inhibiting plasma angiotensin-converting enzyme (ACE) activity (IC_50_ = 198 µg/mL). Exposition of endothelium-intact aortic rings to butein attenuated angiotensin I-induced contraction without angiotensin II-induced contraction [22]. A similar effect was observed by Liu et al. (2003). The authors used three classes (caffeoylquinates, flavan-3-ols, and gallotannins) of tannins from a traditional Chinese herb extract and observed a significant noncompetitive mode of ACE inhibition. Moreover, the authors observed that the ACE activity does not depend on bovine serum albumin addition, but ZnCl_2_ significantly decreased the enzyme activity in methyl containing tannins (methyl 3,4-dicaffeoylquinate and methyl 3,5-dicaffeoylquinate). The in vivo study using spontaneously hypertensive rats (SHRs) confirmed strong hypotensive effects with 10, 20, and 40 mg/kg dose-dependently decreasing blood pressure. The observed effect was stronger than that in the control group using captopril (10 mg/kg) [23].

The anti-inflammatory and vasoprotective effects of curcumin were analyzed by Hernandez et al. (2018). Their in vivo study showed that curcumin treatment (100 mg/kg/day) of recently infected mice by *Trypanosoma cruzi* led to the reduction of myocardial artery inflammation, decrease of the total vessel inflammation score, and increase of cardiac proinflammatory cytokine (IL-6, TNFα) mRNA expression without modifying the bloodstream parasite burden. Moreover, a decrease in ET-1 peptide level was observed in vivo after curcumin therapy and in vitro using human microvascular endothelial cells (HMECs-1). Furthermore, the in vitro study showed that curcumin decreases the release of the vasoactive peptide, reduces [Ca^2+^]_i_ response, and prevents parasite-triggered NFATc1 nuclear translocation, leading to the cytoplasmic accumulation of NFATc1 protein (Figure 1). This suggests that curcumin possesses cardioprotective activity and may abolish the [Ca^2+^]_i_-associated activation of the NFATc1 isoform in vascular endothelial cells infected by *Trypanosoma cruzi* [24].

In vivo studies have shown that pterostilbene improves cardiac function and decreases oxidative stress and inflammation markers (TNFα, IL-1β, myeloperoxidase activity) in rat models of ischemia-reperfusion injury. Moreover, pterostilbene increases Bcl-2 and decreases Bax expression. Thus, it can protect against apoptosis and reduce cardiac inflammation [16]. Pterostilbene also protects vascular endothelial cells against autophagy by stimulation of calcium/calmodulin-dependent protein kinase beta, which activates AMPK and finally inhibits mTOR signaling (Figure 1). In vascular smooth muscle cells, pterostilbene decreases cellular proliferation and regulates Akt kinase, leading to cell cycle progression (Figure 2) [16]. It is noteworthy that in vivo studies have shown that pterostilbene suppressed proinflammatory cytokines (TGFβ), TNFα, IL-1β, and IL-6 in mice, leading to a reduction of high fat-induced atherosclerosis. Furthermore, piceatannol (15–45 mg/kg) treatment in mice caused a decrease in plasma lipopolysaccharides, LDL cholesterol levels, and lipid peroxidation [21].

In atherosclerosis, resveratrol protects against lipid peroxidation and prevents polyunsaturated fatty acid oxidation in low-density lipoprotein. It is possible since resveratrol not only scavenges free radicals and chelate metal ions but also increases the activity of antioxidative enzymes. It is noteworthy that the antioxidant effect was much stronger than the antioxidant activity of α-tocopherol [25].

The effect of resveratrol on blood pressure in vivo and in humans was confirmed by Riche et al. (2014), Thandapilly et al. (2010, and 2013). Resveratrol (2.5 mg/kg/day for 10 weeks) increased mesenteric small artery compliance and reduced wall stiffness in normotensive Wistar–Kyoto (WKY) rats, attenuated arterial compliance in spontaneously hypertensive rats (SHRs), and inhibited extracellular signal-regulated kinase (ERK) signaling [4]. A concentration of 2.5 mg/kg/day for 10 weeks was too low to reduce systolic blood pressure (only 200 mg/kg/day for 4 weeks reduced the blood pressure in SHRs) [26]. In the case of humans, only one study showed that a high dose of pterostilbene, at a concentration of 125 mg twice daily, reduced systolic and diastolic blood pressure [27].

Regarding pellet aggregation, animal and human studies showed that resveratrol (50 µg/mL) inhibits aggregation caused by collagen, epinephrine, and thromboxane probably by the cyclooxygenase (COX)-1 suppression, inhibits the MAPK pathway and phosphoinositide signaling, and activates the nitric oxide/cGMP pathway. Like resveratrol, pterostilbene and gnetol inhibit pellet aggregation, but not that which is induced by thrombin [21]. Moreover, Remsberg et al. 2015 showed that gnetol inhibits adipogenesis in preadipocytes and inhibits COX-1 more strongly than COX-2, which suggests antithrombotic effects [28].

Resveratrol also delays the initiation and progression of atherosclerosis by modification of vascular function, attenuation of lipid accumulation, and modulation of the expression of lipogenesis- and lipolysis-participating genes. Moreover, resveratrol and pterostilbene can decrease the apoptosis of vascular endothelial cells induced by oxidized LDL [21].

In the case of cardiac hypertrophy, resveratrol prevents cardiomyocyte hypertrophy by the activation of NO-AMPK signaling. Moreover, pressure overload-induced hypertrophy may be reduced by resveratrol via a decrease of oxidative stress and upregulation of endothelial nitric oxide synthase (eNOS), leading to an increase in NO production [21]. It is noteworthy that the in vivo study of gnetol and pterostilbene showed the activation of AMPK in isolated neonatal rat cardiomyocytes, leading to ET-1-induced hypertrophy, while another study showed that treating spontaneously hypertensive heart failure rats with 2.5 mg/kg/day of gnetol, pterostilbene, and resveratrol for 8 weeks did not decrease ventricular hypertrophy [21].

Interestingly, human studies using resveratrol in the prevention of primary and secondary cardiovascular diseases are very promising [21]. In a 1-year follow-up study by Tomé-Carneiro et al. (2012), 75 patients were divided into three groups: resveratrol-containing grape extract capsules (8.1 mg/day for the first 6 months and 16.2 mg/day for the next 6 months), grape extract capsules without resveratrol, and placebo capsules. The patients were taking statins to prevent cardiovascular diseases. After 1 year of treatment, the group treated with resveratrol-containing grape extract showed improvement in inflammatory and fibrinolytic status in comparison with the two other groups [29]. Interestingly, the next 1-year study of Tomé-Carneiro et al. (2013) showed that the cardioprotective effect of resveratrol is possible by improving anti-inflammatory response and preventing atherothrombotic signaling. The 75 stable patients with coronary artery disease were divided into three groups: placebo (350 mg/day), resveratrol-containing grape extract (grape phenolics plus 8 mg resveratrol), and conventional grape extract without resveratrol (single dose for 6 months and a double dose for the following 6 months). The study showed an increase of the anti-inflammatory serum adiponectin and a decrease of the thrombogenic plasminogen activator inhibitor type 1 (PAI-1) in the resveratrol-containing group. This suggests that resveratrol may be used in cardiovascular disease treatment [30]. Other human studies showed that resveratrol did not reduce LDL/HDL ratio or affect lipid parameters, such as total cholesterol, LDL, HDL, and triglyceride levels [21].

Scoditti et al. (2012) also analyzed the effect of resveratrol on atherosclerotic vascular disease. Resveratrol (1 µmol/L), like quercetin, stimulates the tubelike differentiation of HUVECs and HMECs-1. Moreover, resveratrol inhibits HUVEC migration and MMP-9 expression and decreases COX-2 expression, without affecting the constitutive expression of COX-1. A decrease of intracellular ROS was also observed after resveratrol treatment (Figure 1) [20].

The ability of resveratrol to decrease intracellular reactive oxygen species levels by inducing autophagy through the AMPK–mTOR pathway was analyzed by Song et al. (2018). The study showed that resveratrol decreased ROS level, caused by palmitic acid treatment, in human aortic endothelial cells (HAECs) by an increase of SOD activity and a decrease of iNOS expression. Moreover, resveratrol significantly “increased the LC3II/LC3I expression ratio and decreased p62 expression in a dose-dependent manner” by a specific AMP-activated protein kinase (AMPK) pathway activator (Figure 1). This suggests that the AMPK pathway may induce a degradation of damaged organelles under metabolic stress (autophagy) and improve endothelial dysfunction. Furthermore, the AMPK–mTOR pathway attenuates in autophagy regulation caused by resveratrol. It is noteworthy that autophagy mediates the resveratrol-induced decrease in ROS, which was confirmed by the silencing Atg5 with siRNA and autophagy inhibitor 3-MA analysis. The in vivo study using C57BL/6 mice showed, as in in vitro studies, that resveratrol increased the L/C3II/LC3I ratio and decreased p62 expression, confirming the ability for autophagy regulation and ROS savaging [31].

The ex vivo effect of resveratrol on the dilator responses of femoral arteries using pathogen-free 4- (young) and 26-month-old (old) male C57BL/6 mice was analyzed by Diaz et al. (2019). The study showed that resveratrol increased dilation to acetylcholine in both mouse age groups and did not affect the sensitivity of smooth muscle cells on NO. Acetylcholine at a concentration range from 10^−9^ to 10^−3^ M was used to induce concentration-dependent dilation in isolated pressurized femoral arteries. Moreover, a decrease of dilator responses after coincubation of vessels with resveratrol, L-NNA, and potassium channel inhibitors (apamin and Tram 34) and no inhibition of acetylcholine-induced dilation after coincubation with resveratrol were observed. Lack of flow-induced dilation at higher flow rates, but not entirely at lower flow rates in vessels of both young and old mice, was also observed. This suggests that resveratrol did not induce further reductions in flow-mediated dilation. A study of eNOS knock-out mice showed that the coincubation in resveratrol and potassium channel inhibitors significantly reduced dilator responses. The in vitro study using human coronary artery endothelial cells (HCAECs) showed that resveratrol treatment increased NO production (Figure 1) at 2 min. During flow conditions, the NO production decreased, and it was not caused by the reduction of cell viability [32].

The biphasic effect of resveratrol on HUVEC cell line survival was analyzed by Posadino et al. (2019). The study showed that resveratrol at a concentration of 1 µM decreases basal intracellular ROS levels and roGFP oxidation state, while at concentrations of 10 and 50 µM, it increases ROS levels and roGFP oxidation state (Figure 1). This suggests that resveratrol acts as an antioxidant agent at a concentration of 1 µM. Moreover, a significant decrease of viability and DNA synthesis at resveratrol concentrations of 10 and 50 µM and a nonsignificant increase of viability and DNA synthesis at a concentration of 1 µM were observed (Figure 1). This suggests a correlation between pro-oxidant effect and cell damage. Furthermore, resveratrol at a concentration of 50 µM increases DNA fragmentation, decreases *Bcl-2* mRNA levels, and increases Bax expression, while at a concentration of 1 µM, no stimulation of DNA fragmentation, an increase in *Bcl-2* mRNA levels, and a low expression of Bax were observed (Figure 1) [33]. This suggests a correlation between pro-oxidant effect, viability, and apoptosis. An analysis of c-myc, which is a key regulator of cell cycle progression and cell proliferation, showed an increased level of c-myc at a low concentration of resveratrol and a high level of the protein at higher concentrations of resveratrol. Moreover, high concentrations of resveratrol “blocked HUVEC cell cycle progression by prompting their accumulation in the G0/G1 or S phase depending on the dosage” and significantly decreased the cyclin D1 level (Figure 1). Thus, c-myc modulation and Bcl-2 expression “may suppress HUVEC proliferation by downregulating cyclin D1 expression ultimately arresting the cells in G0/S phases of the cell cycle.” In the case of PKC level, an increase was observed at a concentration of 1 µM, while a decrease was observed at higher concentrations. It is noteworthy that the increased level of PKC was reversed by the scavenging action of tempol. This suggests that PKC activation by resveratrol depends on ROS levels. Moreover, a broad flavin oxidase inhibitor (DPIO) and a broad PKC inhibitor (CHE) “were able to abrogate the biphasic effect of resveratrol on *c-myc* and *Bcl-2* gene expression and HUVEC proliferation” [33].

The effect of sirtuin family expression on the anti-inflammatory activity of resveratrol on HUVECs was analyzed by Yu et al. (2019). The study showed that TNFα (10 µg/L) significantly decreased cellular viability, while resveratrol (0–40 µmol/L) is nontoxic to the cells. The mix of TNFα and resveratrol (20 µmol/L) showed that resveratrol reversed the side effects of TNFα. Moreover, resveratrol treatment caused more than onefold increase in the expression of SIRT1 to SIRT7 genes, but not in SIRT4, where a decrease in expression was observed (Figure 1). “The mRNA expressions of SIRT1, SIRT2, SIRT5, and SIRT7 were statistically increased in resveratrol pre-treated cells compared with the treated TNF-α cell alone. However, SIRT3, SIRT4, and SIRT6 gene expression had no statistical significance in HUVECs.” Furthermore, siRNA targeted SIRT1, SIRT2, SIRT3, SIRT4, and SIRT5 and reversed the inhibitory effect of resveratrol on TNFα-induced ROS production. This suggests that resveratrol may attenuate oxidative stress in TNFα-induced HUVECs by activating SIRT1, SIRT2, SIRT3, SIRT4, and SIRT5 pathways [34].

The impact of resveratrol and grape extract on HASMC proliferation and gene expression was analyzed by Wang et al. (2006). The authors observed a dose-dependent decrease of growth (50% reduction in proliferation resulted from treatment with 10–25 µM resveratrol) with no impact on cell viability. Resveratrol also had little or no effect on cyclin D1 and PCNA (Figure 2), while a significant increase of p53 expression was observed at a concentration 10 and 50 µM. It is noteworthy that p53 is a transcription factor of several genes (e.g., p21, the cell cycle checkpoint regulator) and heat shock protein 27 (HSP27) responsible for the attenuation of intimal hyperplasia. The expression of p21 was very low, while HSP27 expression significantly increased after resveratrol treatment (Figure 2). Treating cells with resveratrol at a concentration of 50 µM significantly decreased NFκβ expression. Resveratrol treatment also significantly increased quinone reductase I and II (QR1, QR2) in fraction 2, decreased them in fraction 1, and enhanced the partitioning of QR2 in fractions 3 and 4. In the case of eNOS and AIF, enhanced partitioning was observed in fractions 3 and 4, respectively [35].

### 2.3. Phenolic Acids

Scoditti et al. (2012) also analyzed the effect of virgin olive oil polyphenols (oleuropein, hydroxytyrosol) on atherosclerotic vascular disease. The study showed a stimulation of tubelike differentiation of HUVECs and HMECs-1 after 1 h pretreatment with oleuropein and hydroxytyrosol (10 µmol/L) and inhibited HUVEC migration. Interestingly, hydroxytyrosol and quercetin showed the same result. As in the case of quercetin and resveratrol, olive polyphenols also inhibited the MMP-9 expression in endothelial cells and decreased COX-2 expression, without affecting the constitutive expression of COX-1, and decreased the intracellular ROS level (Figure 1). This confirms the cardioprotective effect of olive and wine polyphenols and antioxidant activity [20].

The role of sinapic acid in hypertension and cardiovascular remodeling was analyzed by Silambarasan et al. (2014). The authors observed a decrease in blood pressure, increase in the heart-weight-to-body-weight ratio, and prevention of marked vascular fibrosis in rats treated with sinapic acid at a concentration of 40 mg/kg. Moreover, an increase of antioxidant enzymes (SOD, CAT, GPx), GSH, and aortic nitrite/nitrate levels, as well as higher activity of ACE in the heart and aorta, was observed after sinapic acid treatment. An analysis of cardiovascular function showed that sinapic acid promotes ventricular function, stimulates acetylcholine-induced relaxation in endothelium-intact aortic rings, and restores vasodilatation. Furthermore, the expression of TGFβ and β-MHC was reduced, while eNOS expression was increased after sinapic acid treatment. The in vitro study using the immortalized endothelial hybrid cell line EA.hy926 showed that sinapic acid did not affect cellular viability (up to 100 µM) and increased viability against H_2_O_2_-induced oxidative stress (1 and 10 µM). It is noteworthy that the evaluation of total ROS and NO levels showed that sinapic acid significantly protects cells from ROS generation and increases NO level (Figure 1) [36].

### 2.4. Polyphenol Containing Plant Concentrates, Extracts, and Fractions

Other human studies showed that cocoa polyphenols (flavanols, flavonols, anthocyanins, flavones, flavanones, and phenolic acids) decrease LDL oxidation and lipid peroxidation biomarkers (F2-isoprostane). Moreover, dark chocolate consumption decreases serum LDL cholesterol concentrations and plasma triglyceride and increases the susceptibility of LDL to oxidation and plasma high-density lipoprotein (HDL) levels. Moreover, the role of flavonoids in chocolate and cocoa included stimulation of antioxidant effects and increase of antioxidant activity, modest anti-inflammatory effect, inhibition of platelet aggregation (by cellular eicosanoid synthesis downregulation), and reduction of biological membranes’ lipid peroxidation. Unfortunately, there are also meta-analysis studies showing no effect on LDL concentrations after chocolate, cocoa, and flavonoids consumption [37].

Interestingly, the fixed meta-analysis performed by Peters et al. (2001) suggest about 11% decreased incidence of myocardial infarction after drinking three cups of tea per day [38].

The effect of the polyphenol-rich ethanol extract of bee pollen (EPP) on ACE level was analyzed by Rzepecka-Stojko et al. (2017 and 2018). The in vivo study, which used C57BL6 ApoE-knockout mice, showed a lower ACE level (152 ng/mL) in animals on a standard diet supplemented with bee pollen extract at a dose of 1 g/kg BM. In contrast, ACE level increased to 161 ng/mL in animals on a high-fat diet [39]. Similar results were obtained in the in vivo study using C57BL6 mice. Animals on a high-fat diet showed an increase in ACE level up to 145 ng/mL while supplementing a high-fat diet with EEP, resulting in a decrease of ACE level to 121 ng/mL [40].

Wang et al. (2006) also analyzed the impact of a grape extract on HASMC proliferation and gene expression. The extract also dose-dependently decreased cell proliferation. The extract at a concentration of 0.02% showed similar results as resveratrol at a concentration of 25 µM. In contrast to resveratrol, the extract altered the distribution of cytochrome oxidase (OX II) in fractions 3 and 4 of the treated cells (Figure 2) [35].

Persson et al. (2009) also analyzed the impact of *Vaccinium myrtillus* extract on ACE in human HUVECs. The study showed a decrease in cellular ACE activity after *Vaccinium myrtillus* extract treatment (Figure 1). Moreover, the extract did not affect cellular viability [16].

## 3. Discussion

The increasing number of deaths caused by cardiovascular diseases requires new and more effective methods of prevention and/or treatment. This can be achieved by polyphenol treatment since polyphenols possess anti-inflammatory, antimicrobial, antioxidant, antithrombotic, cardioprotective, and vasodilatory effect activities. It is noteworthy that those effects were observed and confirmed by several research groups using endothelial cells and cardiomyocytes not only in vitro but also in vivo.

The effect of flavonoids, nonflavonoids, and phenolic acids on human endothelial cell lines—EA.hy926 [36], HAECs [31], HCAECs [32], HMECs-1 [20,24], and HUVECs [15,17,20,21,33,34]—were analyzed by different groups.

Silambarasan et al. (2014) showed that sinapic acid attenuates hypertension and improves cardiovascular function. The protective action of sinapic acid is connected with increasing NO level and decreasing ACE activity through antioxidant potential. It is possible since agents with antioxidant properties increase NO level and improve the regulation of vascular tone, while a decrease of NO level may lead to a volume-dependent increase of blood pressure. Moreover, EA.hy926 cell assay showed that sinapic acid did not affect cellular viability, increased viability against H_2_O_2_-induced oxidative stress, and significantly protected cells from ROS generation and increased NO level. Since polyphenol reduced oxidative stress and myocardial fibrosis observed in hypertensive rats, this suggests its therapeutic potential in hypertensive heart disease treatment [36].

Hernandez et al. (2018) showed that curcumin reduces cardiac vasculopathy in mice by the inflammation of heart vessels and vascular permeability reduction. Moreover, curcumin decreases ET-1 level and proinflammatory cytokines (IL-6, TNFα) in HMECs-1. ET-1 is an important contributor to vascular compromise, playing a key role in the development of vascular disruption. ET-1 is also a proinflammatory cytokine that promotes vasoconstriction, vascular permeability, platelet aggregation, and heightened production of adhesion molecules and inflammatory agents. The obtained results suggest that curcumin significantly impairs ET-1 secretion from *Trypanosoma cruzi*-infected vascular endothelium by blocking the Ca^2+^/calcineurin/NFATc1 cascade. Thus, the anti-inflammatory and cardio-/vasoprotective activity of curcumin makes it a potential tool in the treatment of cardiomyopathy [24].

Song et al. (2018) showed that “resveratrol inhibits intracellular ROS production by inducing autophagy via the AMPK–mTOR pathway” in HAECs. It is possible by increasing the LC3II/LC3I ratio, suggesting a stimulation of autophagic flux, which mediates the antioxidant effect of resveratrol. Thus, autophagy induction by resveratrol may cause vascular protection. Moreover, the protective effect of resveratrol may be related to the AMPK pathway. It is noteworthy that AMPK is the upstream target for mTOR, and the AMPK–mTOR pathway additionally normalizing ROS production by regulating mitochondrial biogenesis prevents endothelial apoptosis and inhibits vascular inflammation. Thus, this suggests the potential role of resveratrol in cardiovascular disease treatment. It is noteworthy that the in vitro results were confirmed by in vivo assays [31]. Furthermore, pterostilbene stimulates calcium/calmodulin-dependent protein kinase beta, which activates AMPK and finally inhibits mTOR signaling, leading to the protection of vascular endothelial cells against autophagy [21].

Diaz et al. (2019) showed that ex vivo exposure to resveratrol enhances acetylcholine-induced dilation, but not flow-mediated dilation in HCAECs. This suggests that resveratrol may impair “flow-mediated endothelial mechano-transduction at low shear stress but potentiate the release of acetylcholine-induced endothelial-derived mediators, by stimulating the endothelial-derived hyperpolarizing factor (EDHF) and NO pathways.” All observations suggest that resveratrol may be used in a therapeutic strategy to modulate the dilator function [32].

Zhao et al. (1999) showed that quercetin inhibits endothelin 1 and tissue plasminogen activator release and stimulates prostacyclin (PGI_2_) release from endothelial cells. A decrease of endothelin 1 may be caused by the inhibition of protein kinase C (PKC) or an increase of nitric oxide (NO). Both mechanisms are possible since PKC is activated by phospholipase C and PKC regulates the expression of endothelin 1, and NO inhibits endothelin 1 release, and quercetin can increase NO release. An increase of prostacyclin release may be caused by the inhibition of cyclooxygenase (CO), which metabolizes arachidonic acid. Moreover, the ability of flavonoids to perform free radical scavenging may lead to the inhibition of polyunsaturated fatty acid peroxidation, which is required for the activation of CO. On the other hand, the release of PGI_2_ by endothelial cells enables the inhibition of platelet aggregation, which is desirable in healthy (not atherogenic states) organisms. In atherogenic states, a decrease of PGI_2_ is a disadvantage since it would let vasoconstrictors (endothelin 1 and angiotensin II) determine the capacity of the artery to maintain its lumen. A decrease of tissue plasminogen activator (t-PA) may be caused by plasminogen activator inhibitor (PAI-1) inhibition. PAI-1 is the main physiological inhibitor of t-PA. PAI-1 inhibition by flavonoids is possible by PKC activity inhibition and a reduction in Ca^2+^ elevation [19]. All these observations suggest and confirm the cardioprotective role of polyphenols. Persson et al. (2009) showed that *Vaccinium myrtillus* extract inhibits ACE in HUVECs more strongly than myrtillin chloride or anthocyanidins. It is possible since bilberry contains anthocyanins (glycosides of anthocyanidins), anthocyanidins, flavonol, quercetin, stilbene resveratrol, ferulic acid, and coumaric acid. It is noteworthy that quercetin at a concentration of 30 mg/kg strongly inhibits ACE [16]. Scoditti et al. (2012) showed that red wine and virgin olive oil polyphenols, such as oleuropein, hydroxytyrosol, resveratrol, and quercetin, inhibit endothelial tube formation in HUVECs and HMECs-1 and decrease cell migration and suppress of ROS production by NFκβ-dependent MMP-9 and COX-2 expression in HUVECs. This suggests the anti-inflammatory and antiangiogenic activities of polyphenols and confirms their beneficial effect on cardiovascular disease [20]. Negrão et al. (2013) showed that catechins effect the angiogenic process, viability, proliferation, migration, invasion, and capillary differentiation capacity in HUVECs and HASMCs. The observed increase of both cell line viabilities may be caused by a decrease of apoptosis after catechin treatment. A decrease of proliferation suggests that catechin “stabilizes HUVEC and HASMC in the absence of a prominent proangiogenic environment.” The anti-inflammatory effect of catechins was confirmed by a decrease of NFκβ amount, as well as TNFα and NO levels [30]. Posadino et al. (2019) showed that low concentrations of resveratrol are beneficial. Resveratrol at a concentration of 1 µM decreases ROS and apoptosis (decrease *Bax*, increase *Bcl-2*) and increases cellular viability, DNA synthesis, and cell cycle regulation genes (*c-myc*, *ODC*, and *cyclin D1*) of HUVECs. In higher concentrations, they are harmful rather than beneficial since cell cycle arrest, apoptosis, DNA fragmentation, decrease of DNA level, cellular viability decrease, and ROS increase were observed [33]. Yu et al. (2019) showed that resveratrol regulates the transcriptional and translational levels of SIRT1, SIRT2, SIRT5, and SIRT7 in HUVECs. It is important since SIRT1 regulates the modification of histones and many nonhistone proteins important for apoptosis, inflammation, and stress resistance [34].

Taking into account all of the results, it is possible to confirm the cardio- and/or vasoprotective effects of polyphenols on endothelial cells by the

- decrease of ACE, ET-1, ROS production, endothelial tube formation, migration, endothelin 1, and tissue plasminogen activator release;
- stimulation of acetylcholine-induced endothelial-derived mediator release, autophagy, NO, DNA synthesis, and cell cycle regulation;
- increase of viability; and
- regulation of sirtuins.

It is very important to take into consideration the concentration of used polyphenols since their observed positive effect on low concentration may cause an opposite effect on higher concentration, as Posadino et al. (2019) observed. The impact of polyphenols on endothelial cells is shown in Figure 1.

The effect of grape extract, flavonoids, and nonflavonoids on human endothelial cell lines—HASMCs [17,18,21,35] and monocytes [7]—was analyzed by different groups. Wang et al. (2006) showed that resveratrol and grape extract decreased cell proliferation. Moreover, resveratrol did not have an impact on cellular viability, cyclin D1, p21, and PCNA expression; significantly increased p53 expression; and decreased HSP27 expression in HASMCs. Interestingly, analysis of fractions showed opposite results. This suggests that in the regulation of HASMC growth and gene expression by SGE and resveratrol, different mechanisms are involved [35]. Olszaniecki et al. (2008) showed that only kaempferol, not resveratrol, can inhibit ACE, which is probably caused by their difference in chemical structures [18]. Moreover, regulation of Akt kinase and a decrease of cellular proliferation was observed after vascular smooth muscle cell treatment by pterostilbene [21]. A decrease of proliferation was also observed after HASMCs by catechin treatment [36]. Atrachimovich et al. (2019) showed that quercetin binds to the E2F DNA motif of human monocyte THP-1 cells and regulates the expression of 36 different genes related to cell cycle and cell development [7]. The impact of polyphenols on aortic cells and monocytes is shown in Figure 2.

Most promising are the in vivo results showing the effect of cardioprotective polyphenols in studies performed using animals [4,17,21,22,23,24,25,26,31,39,40] and humans [21,27,28,29,30,35,36,37,38].

Kang et al. (2003) showed in an in vivo study that butein decreases blood pressure via the inhibition of ACE activity. It is possible since the aromatic hydroxyl groups of polyphenol (butein) may inhibit ACE activity by the generation of zinc chelate complexes in the active center of ACE [22]. This was confirmed by Liu et al. (2003) since the supplementation of ZnCl_2_ showed a decrease of about 30% ACE inhibition caused by caffeoylquinates. This suggests that the chelation of zinc ions by polyphenols may be responsible for the inhibition of ACE activity. Moreover, a decrease in blood pressure was observed in an in vivo study [23]. Thandapilly et al. (2010 and 2013) showed that resveratrol increases mesenteric small artery compliance and reduces wall stiffness and inhibits ERK signaling in vivo [4]. Moreover, resveratrol at a concentration of 2.5 mg/kg/day for 10 weeks is not enough to reduce systolic blood pressure [25]. Furthermore, the in vivo study showed that catechins affect physiological angiogenesis and pathological angiogenesis, presenting higher VEGF-A stimulation [17]. Hernandez et al. (2018) showed that curcumin reduces myocardial artery inflammation and the total vessel inflammation score and increases cardiac proinflammatory cytokine (IL-6, TNFα) mRNA expression. Moreover, as in the case of the in vitro study, a decrease in the ET-1 peptide level was observed [24]. Moreover, Akinwumi et al. (2018), in a review, suggested the cardioprotective activity of stilbenoids (astringin, gnetol, piceatannol, and pterostilbene) since a decrease in mortality rate, Bax expression, TGFβ, oxidative stress, inflammation markers (TNFα, IL-1β, myeloperoxidase activity), lipopolysaccharides, LDL cholesterol levels, lipid peroxidation, and lactate dehydrogenase levels and an increase of Bcl-2 and nitric oxide were observed in rats and mice in vivo. Moreover, a different effect on hypertrophy induction was observed, depending on the used polyphenols [21]. Song et al. (2018) showed that resveratrol regulates autophagy and ROS scavenging in vivo [31]. It is noteworthy that the in vivo studies performed by Rzepecka-Stojko et al. (2017 and 2018) confirmed the ability of polyphenols to inhibit the activity of ACE [39,40]. This also confirms the results of the in vitro studies of Persson et al. (2009) and Olszaniecki et al. (2009), as well as the cardioprotective effect of polyphenols since EEP prevents and inhibits the development of atherosclerotic changes and modulation of the renin–angiotensin–aldosterone system.

Interestingly, animal and human studies have shown that resveratrol inhibits pellet aggregation, phosphoinositide signaling, MAPK, and nitric oxide/cGMP pathways in vivo. Similar results showed pterostilbene and gnetol but not in pellet aggregation induced by thrombin, whereas resveratrol prevents primary and secondary cardiovascular diseases but does not reduce LDL/HDL ratio or does not affect lipid parameters [21]. Moreover, Remsberg et al. 2015 confirmed gnetol antithrombotic effects since it inhibits COX-1 more strongly than COX-2 [28]. In the case of pterostilbene, Riche et al. (2014) showed that reduces systolic as well as diastolic blood pressure [27]. The study of Tomé-Carneiro et al. showed that resveratrol-containing grape extract improves the inflammatory and fibrinolytic status of patients [29], while the second study showed atherothrombotic signaling prevention by resveratrol treatment owing to the decrease in patients’ PAI-1 and the increase in the anti-inflammatory serum adiponectin [30]. Interestingly, the results showed an analysis of cocoa polyphenols (flavanols, flavonols, anthocyanins, flavones, flavanones, and phenolic acids) [37] and tea polyphenols [38]. In the first case, there was a decrease in LDL oxidation, LDL cholesterol, plasma triglyceride concentration, F2-isoprostane, and HDL levels. Additionally, a stimulation of antioxidant activity, modest anti-inflammatory effect, inhibition of platelet aggregation, and biological membrane lipid peroxidation reduction were observed as a potential cardioprotective effects [37]. It is noteworthy that Peters et al. (2001) showed that drinking 3 cups of tea per day decreases myocardial infarction incidence [38].

Despite the potential positive cardioprotective effects of polyphenols, any health claims should not be made unless long-term studies result in measurable health benefits in tested populations. In vitro studies should be used only as an entry point for future clinical studies, not as a final argument of their everyday use.

## Figures and Tables

**Table 1:** Summary number of death cases and percentage of deaths in the world and Europe.

	Cardiovascular Diseases	
	Number of Death Cases	Deaths (%)
World Health Organization [11]	17.9 million (globally)	31.0 (globally)
Eurostat statistics [12]	1.8 million *	35.7 *
European Heart Network [13]	3.9 million ** and 1.8 million *	45 ** and 37 *

* in the European Union; ** in Europe.

**Table 2:** Summary number of stroke death cases per 100,000 population in selected countries.

Country	Stroke Death Cases per 100,000 Population in 2017 [15]
Canada	35.1
Australia	42.0
USA	43.2
Japan	46.1
Korea	56.1
Poland	71.0
Brazil	81.5
Turkey	97.2
South Africa	140.7
Russia	234.4
